# Timbre-induced pitch shift from the perspective of Signal Detection Theory: the impact of musical expertise, silence interval, and pitch region

**DOI:** 10.3389/fpsyg.2014.00044

**Published:** 2014-01-31

**Authors:** Allan Vurma

**Affiliations:** Department of Musicology, Estonian Academy of Music and TheatreTallinn, Estonia

**Keywords:** Signal Detection Theory, pitch shift, pitch discrimination, pianists, violinists, timbre, memory, pitch region

## Abstract

The paradigm of Signal Detection Theory (SDT) was used to analyze the ability of professional pianists (*N* = 16) and string players (*N* = 15) to discriminate small F0 differences between consecutive musical tones, presented in pairs, with identical and with different (bright and dull) timbres. The sensitivity (*d*′) and response bias (*c*) were heavily dependent on the timbral arrangement of the pairs of tones (the “comparable tones”), which can be interpreted as the influence of timbre-induced pitch shift on F0 discrimination. The participants were somewhat biased to “miss” signals when comparable tones had identical timbres and to make “false alarms” when the tones had different timbres. The *d*′ was lowest when the tones with a lower F0 in those stimulus-pairs containing tones with different timbres had a brighter timber, and highest when both tones had bright timbre. On average, the string players had a somewhat higher *d*′ and their perception was slightly less influenced by timbre-induced pitch shift when compared to the pianists. Nevertheless, the dependence of *d*′ and *c* on the timbral arrangement of the tones was registered in the case of all the participants at all the investigated pitch regions around D#3, D4, and C#5. Furthermore, the presence of a silence of 3.5 s—a silence interval—between the tones to be compared had an impact on both *d*′- and *c*-values as well as on the degree of vulnerability to timbre-induced pitch shift.

## Introduction

The ability to play or sing in tune (i.e., to use correct pitches) is one of the most important qualities of the professional musician. According to the definitions of ANSI (1994) “pitch is that auditory attribute of sound according to which sounds can be ordered on the scale from low to high.” In Western music it is generally expected that all the steps of the musical scale on which a piece of music is based should be performed at consistent pitches, regardless of the musical instrument used or the location of the note in the score. Nevertheless, the determination of the exact numerical values of the parameters which define the correct pitch of a certain note may appear ambivalent.

On one hand, musicians have agreed internationally that the value of the fundamental frequency (F0) of A4 is set at 440 Hz and that the F0-s of the rest of the scale steps should be calculated from that reference by means of certain formulas (e.g., in the equal tempered chromatic scale the F0 of adjacent scale steps should differ by the factor ${}^{12}\sqrt{2}$). From this perspective it would be logical to assume that pitch in music should be directly linked to the F0 of the particular tone. This standpoint is also supported by the fact that the presence or absence of beats^1^ is often used by musicians as a guide when they have to tune two simultaneous tones to the same pitch (the cessation of beats indicates that the F0-s of two tones are identical).

On the other hand, several investigations have shown that pitch and some other parameters of sound, e.g., its timbre, interact with each other. For example, in the recent experiments of Vurma et al. (2011), a timbre-induced pitch shift with an average magnitude of about 15–20 cents was recorded in the perception of musicians as well as non-musicians when the pitches of consecutive viola and trumpet tones or viola and sung tones (tenor voice) presented in pairs were compared. The brighter trumpet and tenor voice tones were perceived as being slightly higher in pitch than the duller viola tones at the same F0. Parncutt (2013) has referred to the phenomenon where different sensory domains interact as “weak synesthesia.” The interaction of pitch and timbre has also been reported by Chuang and Wang (1978), Wapnik (1980), Terhardt et al. (1982a,b), Melara and Marks (1990), and Russo and Thompson (2005). This implies that tuning which is based on the subjective pitch comparison of two tones may in certain cases be in conflict with tuning based on some theoretical *F*0-value (Vurma et al., 2011).

Besides the timbral difference, the difference in the sound pressure level (SPL) of the tones has also been reported to bias subjective pitch impression (e.g., see Stevens, 1935; Terhardt, 1974; Verschuure and van Meeteren, 1975). Generally, the pitch of pure tones below about 2 kHz tends to decrease and that above 4 kHz to increase with increasing SPL levels, but the effect is less pronounced in the case of complex tones.

The aim of this paper is to investigate the phenomenon of timbre-induced pitch shift from the perspective of Signal Detection Theory (SDT). This statistical method allows us to determine the sensitivity (*d*′) of participants to discriminate “signals” from “noise” independently of the response bias (Macmillan and Creelman, 1991). The bias (*c*) shows whether the person tends to make “false alarms” (erroneous reports about the presence of signal) or to “miss” signals.

We are here using SDT in order to describe the ability of professional musicians to detect whether the pitch of two consecutively played musical tones is different or not. In this context “signals” are the tone-pairs where the F0-s of tones are different and “noise” the tone-pairs containing tones with identical F0-s. We are seeking to refute the null hypothesis that the pitch of a tone is determined solely by its F0. If the null hypothesis holds, the values of *d*′ and *c* should not depend on whether the two tones in a pair have similar or different timbres or on the timbral order of their succession at given F0-s of the tones. In this paper we are not interested in the intervallic values of timbre-induced pitch shift, but only in its influence on the values of *d*′ and *c*. The second goal of our research is to ascertain whether or not any additional factors such as (1) the type of a person's musical expertise, (2) the presence or lack of a brief silence interval between comparable tones, and (3) the pitch region of the tones have any impact on the corresponding parameters.

The particular musical expertise of the person—more specifically, the type of musical instrument which has been played and practiced by a musician over an extended period of time—can influence his/her musical abilities, including pitch perception. For example, a substantial part of the early training of violinists includes the training of good intonation (Kanno, 2003), and violinists as a group have a reputation among other musicians for excelling over their colleagues in tasks connected with intonation. This frequent opinion is supported by the research results of Geringer (1978), which showed that string players deviated less from equally tempered scale step values in the production of the ascending scale than vocalists, wind instrumentalists or keyboard players (pianists intoned the scale with their voice, other musicians used their instrument). In Geringer's research, violinists also proved better than other musicians in tasks where the perception of intonation was investigated. Micheyl et al. (2006) reported similarly that in pitch discrimination, the mean difference limen (DL) for both pure and complex tones was considerably lower for string players than it was, for example, for pianists. This tendency may be caused by the more frequent necessity for string players to make tiny pitch adjustments during performance when compared, for example, to pianists, as the pitch of keyboard instruments is completely determined by the key that is pressed. String players also use the cessation of beats between concurrent tones frequently as a guide for playing in tune (Kanno, 2003), a skill which is not required in piano playing.

Another relevant factor for good performance in intonation tasks may also be the person's familiarity with a particular sound timbre. For example, Greer (1970) reported that much larger mismatches in intonation occurred when brass players played together with an unfamiliar timbre such as an oscillator or organ, compared to a situation where the more familiar timbre of wind instruments was used.

Auditory perception always involves memory mechanisms (Zatorre, 2001), and the presence or lack of a silence interval between comparable tones may influence how these memory mechanisms are involved. Memory can be addressed as three functional processes which work on time frames with different lengths: echoic memory, short-term memory, and long-term memory (for an overview see: Snyder, 2000). At the level of echoic memory, which usually decays in less than 1 s, the sensations are not categorized in any way and they persist as raw continuous sensory data. Short-term memory lasts on average 3–5 s and its contents are no longer primarily raw sensations, but also include activated categorized memories from long-term memory. Recording the information into the long-term memory requires repetition over longer periods and/or elaboration, and the information is stored in the form of conceptual categories (Snyder, 2000). In our experiment such elaboration could include the process of separating pitch information from timbral information by the nervous system. Therefore, we may hypothesize that the timbral impact on pitch perception is smaller when a silence interval is inserted between the comparable tones, giving more time for such processing.

The pitch region of comparable tones may also be a relevant factor for the results of our research, as the frequency DL depends on frequency. In the case of pure tones it is best at mid frequencies of around 500 Hz (Sek and Moore, 1995). In complex tones the extent of timbre-induced pitch shift may possibly depend on the shape of the spectral envelope of tones and on the location of energy peaks in the spectrum. For example, Chuang and Wang (1978) hypothesized that the frequency region between 200 and 500 Hz largely determines the magnitude of the pitch shift of a vowel sound.

## Methods

### Participants

A perception test was administered to 16 pianists (7 male, 9 female) and 15 string players (6 male, 9 female). Of these 31 professional musicians, 29 were the students at the Estonian Academy of Music and Theatre (average age 23, Min 17, Max 27), one violinist was a member of the national state symphony orchestra (age 30) and one pianist was a teacher/accompanist at a music school (age 46). Of the string players, 13 were violinists, 2 were viola players and one was a cellist. All the participants had started their music studies in their early childhood at ages between 5 and 7. All the string players had also studied the piano as the obligatory second instrument of their curriculum. Some of the pianists had also taken lessons on some other instruments: violin (2), conducting (2), trumpet (1), and French horn (1). Five pianists and two string players claimed to possess absolute pitch (AP), although this was not checked. None of the participant had reported any medical hearing problems. This randomly selected sample was representative of all the piano and string students at this music academy, as it included about one third of all the students from the relevant disciplines. All the participants were paid for participating in this study.

### Perception test and stimuli

The perception test consisted of 528 stimulus-pairs of two successive musical tones; each tone was about one second long. Participants had to say whether the pitch of these tones was the same or different. The paradigm of the experiment included four variables: (1) The F0 difference of the tones ΔF0^2^ (which was either zero, or plus or minus 20, 40, or 60 cents), (2) the timbre of the tones and the order of their timbral succession (either bright–dull, dull–bright, bright–bright, or dull–dull), (3) the presence or lack of a 3.5 s silence interval between the tones, and (4) the pitch region of the tones (around either D#3, D4, or C#5).

All the stimuli were created from recordings of the tones of a natural viola by manipulating their parameters with the help of the sound editing program WaveLab 5. These recordings were made in an anechoic chamber in the Wendell Johnson Speech and Hearing Centre at the University of Iowa with a single Neumann KM 84 cardioid condenser microphone, Mackie 1402-VLZ Mixer and Panasonic SV-3800 DAT Recorder at sampling rate 44.1 kHz. (The sounds are publicly downloadable from the institution's database at http://theremin.music.uiowa.edu/MIS.html.)

For the location of the three pitch regions we chose the octaves which are used most frequently in Western music, which lie inside the stave of the treble and bass clef and which include the middle part of the human singing voice range. The *F*0-value for each pitch region's center was chosen randomly by using a random number generator—our intention was to minimize possible tonality-based associations over different stimulus-pairs which might arise while carrying out the test. We then took from the database mentioned above three bowed tones on D#3, D4, and C#5, whose F0-s were the closest matches with the corresponding random numbers.

**Figure 1:**
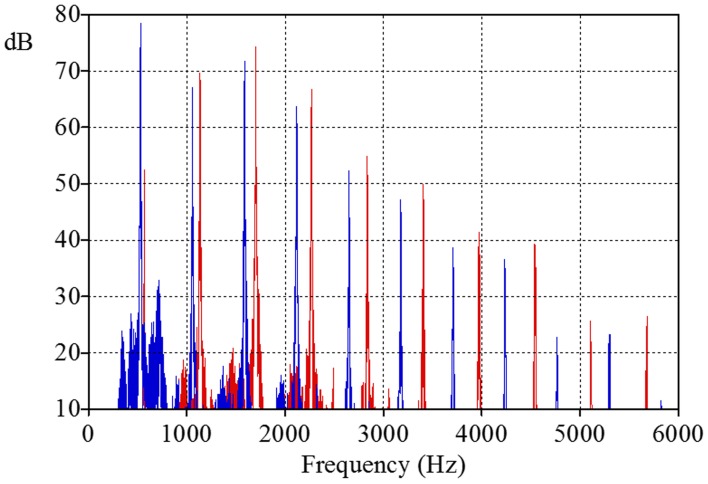
**The spectra of stimuli with bright (see red harmonics) and with dull timbre (see blue harmonics) at pitch region around C#5**.

We then modified the timbre of these tones in two different ways with the help of a sound equalizer in order to obtain stimuli with bright and dull timbral variants. The only difference between the sounds with these different timbres lay in the relative level of their fundamental spectral components: the F0 partial was the strongest component in the case of the tone with the dull timbre, but the same spectral component was about 25 dB weaker in the case of the tone with the bright timbre^3^ (see Figure 1).

**Figure 2:**
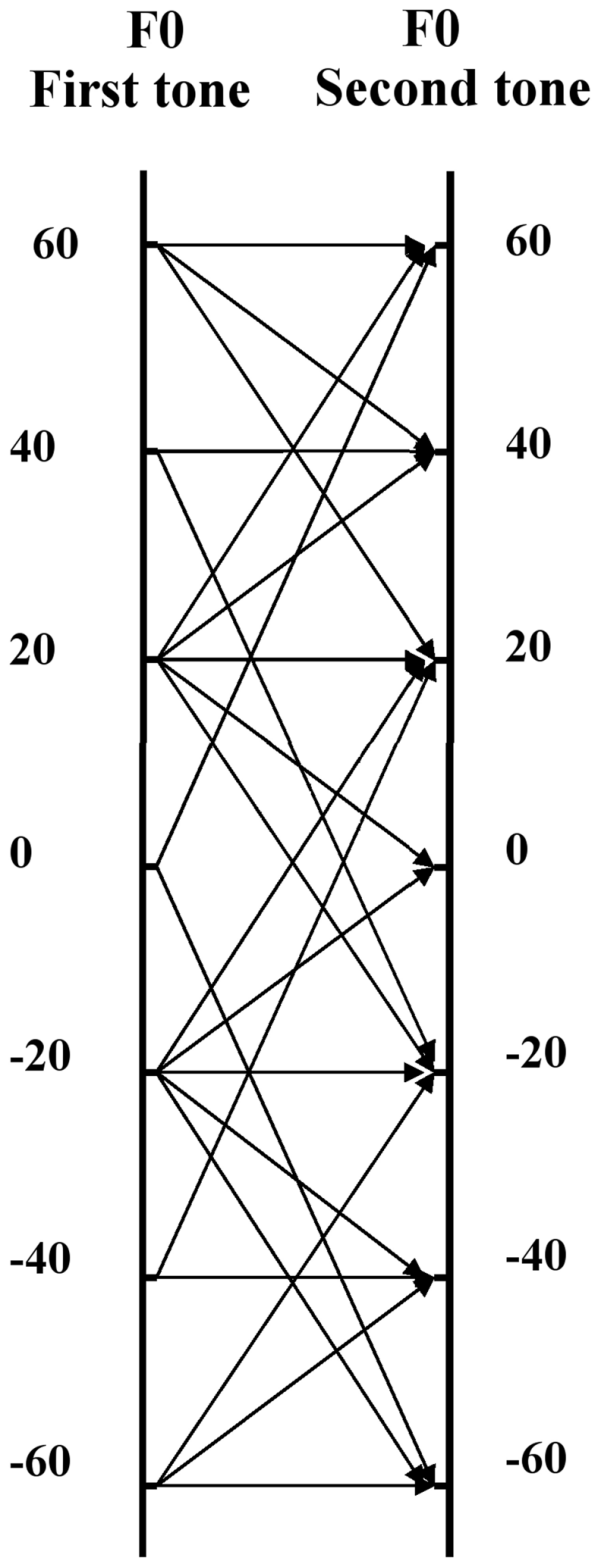
**The schema of 22 F0 combinations (see thin lines with arrows) which were used for creating the tone-pairs with 0 cents, plus and minus 20 cents, 40 cents, and 60 cents F0 difference**. The two vertical lines correspond to the F0 scale of the first and second stimulus. The numbers at these lines show the relative F0 distance of the corresponding stimulus from the center of the pitch region (in cents).

The viola tones used in our experiment had a small random instability of F0 (not exceeding 3 cents), which is typical for the sounds produced by natural musical instruments. The average *F*0-values of the stimuli which corresponded to the middle of the relavant pitch regions were set, with the help of a pitch correction tool, at 158.7, 300.7, and 548.1 Hz (these were the values generated by the random number generator). As the pitch correction used was small, we may consider that its impact on the timbre of stimuli was negligible (with regard to pitch correction methods see also Vurma et al., 2011)^4^. For the creation of the tone-pairs with different Δ*F*0-s, first of all the sets of tones consisting of seven F0 variants were created for each pitch region (again, with the help of the pitch correction tool). The F0 increment of the tones in each set was 20 cents. These seven tones were then combined pairwise in different ways in order to get seven Δ*F*0 variants. As various F0 combinations were used for the same Δ*F*0, the total number of F0 combinations was 22 (see Figure 2). As the results of a brief pilot test showed a much smaller impact on the effects which are investigated in this work at Δ*F*0-s >50 cents, and in order to keep the load for the participants within reasonable limits, we used six tone-pairs for the Δ*F*0-s of 0, 20, and 40 cents (the last two of which included three tone-pairs with the Δ*F*0 direction “up” and three with the Δ*F*0 direction “down”), but only four tone-pairs for the Δ*F*0 of 60 cents (which included two tone-pairs with the Δ*F*0 direction “up” and two with the direction “down”). As a final step the SPLs of all the sounds were equalized; the perceived volume of all the tones was also almost the same. To summarize, we created 22 F0 combinations × 4 timbral variants × 2 sets (with and without a silence interval between the tones) × 3 pitch regions = 528 stimulus-pairs.

The perception test was administered to the participants using a Praat5 software listening experiments module diotically through Sennheiser HD590 headphones at about 90 dB SPL (the SPL was checked by using a B&K head and torso simulator type 4128C with a flat frequency response). The intention was to use the SPL level which is commonly experienced by the musicians themselves when playing their instrument rather than by a member of the audience in a concert hall. It is known that for the position of violinists, the actual SPL ranges of their instrument measures between 84 and 103 dB (Benninger, 1994). The unevenness of the frequency response of the headphones at the spectral band of the tones used was <3 dB.

The participants had to give their responses to each pair by pressing the appropriate button (“the same” or “different”) on the laptop (HP Compaq 6715b) screen. The participants were asked to make their decisions on the basis of pitch only and to ignore the timbral information. Each stimulus-pair was played only once, but the response time was not limited. The next stimulus-pair followed about 1 s the response to the previous pair. After each 10 pairs the participant could take a rest as long as he/she wished. It took between 65 and 85 min for the participants to complete the whole test. No training sessions were conducted and no feedback was given to the participants during the test. The calculation of *d*′, *c* and other statistical parameters was accomplished with the help of a web-based online calculator accessible from: http://www.computerpsych.com/Research_Software/NormDist/Online/Detection_Theory (last visit 27.07.2013).

## Results

Fifteen pianists and 16 string players gave altogether 16368 replies (the sum of all “hits,” “misses,” “false alarms,” and “correct rejections”). Of these replies 11904 were ratings to “signals” (the sum of “hits” and “misses”); the remaining 4464 replies were ratings to “noise” (the sum of “correct rejections” and “false alarms”).

### General group-sensitivity of pianists and string players over all test variables

First, we pooled the replies separately for pianists and string players over all the test variables and calculated the statistical SDT analysis parameters (see Table 1). The group-sensitivity of string players (*d*′ = 1.39) as well as the rate of their total correct replies (75.9%) was somewhat higher than the corresponding values for pianists (*d*′ = 1.08 and 69.9%). The values of *c*, which were close to zero in both groups, indicated no substantial bias either toward “missing” the “signals” or toward making “false alarms.”

**Table 1:** **Statistical group-parameters of the SDT analysis of pianists and string players (the replies were pooled over all test variables)**.

	**Pianists**	**String players**
Hits	4238	4388
Misses	1906	1372
False alarms	641	535
Correct rejections	1663	1625
Signals	6144	5760
Noise	2304	2160
*d*′	1.08	1.39
*d*′ *standard error*	0.03	0.04
*c*	0.05	−0.02
*c standard error*	0.02	0.02
“Hit” rate	0.69	0.76
“False alarm” rate	0.28	0.25
Percent correct	69.9	75.9
Percent correct *standard error*	0.98	0.94

*Signals—the stimulus pairs with different F0-s; Noise—the stimulus pairs with equal F0-s*.

### The impact of the timbral arrangement of the stimuli

**Figure 3:**
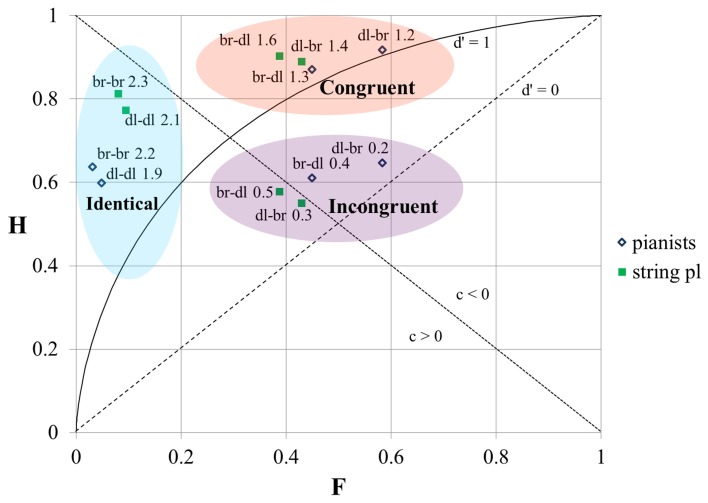
**The results of the SDT analysis of the responses of pianists and string players at different timbral orders of stimuli**. H, “hit” rate; F, “false alarm” rate; br, bright timbre; dl, dull timbre; the numbers on the graph correspond to *d*′-values. For the diagonals and the bowed curve see explanation in the text.

The results of the SDT analysis of the replies of pianists and string players sorted by the different timbral arrangements of the stimuli are shown in Figure 3. The horizontal axis on the graph shows the “false alarm” rate (F) and the vertical axis shows the “hit” rate (H). The broken isosensitivity (*d*′ = 0) diagonal line rising from the lower left corner divides the plot into two sections. Above that line the value of *d*′ > 0 (the “hit” rate exceeds the “false alarm” rate, and the “correct rejection” rate exceeds the “miss” rate). Below that line the value of *d*′ < 0 (the “false alarm” rate is higher than the “hit” rate and the “miss” rate is higher than the “correct rejection” rate). The shape of the isosensitivity line turns into bowed curves at *d*′ > 0. The graph shows one isosensitivity curve which corresponds to *d*′ = 1 (at *d*′ = 1, and when *c* = 0, both the “hit” rate and the “correct rejection' rate are equal to 0.69). The other broken diagonal line falling from the upper left corner also divides the graph into two areas. Above this line *c* < 0 (in this area “false alarms” prevail over “misses”); below this line *c* > 0 (“misses” prevail over “false alarms”).

We can see that our data-points are positioned at three distinct loci. These locations are similar for both groups—for the pianists as well as for the string players. The data-points which are located in the upper left corner correspond to the responses to stimulus-pairs containing tones with identical timbres (bright–bright or dull–dull). The value of *d*′ here is the highest, as the “hit” rate is high and the “false alarm” rate is low. The value of *c* > 0 indicates that the participants were biased to “miss” “signals.”

The data-points in the upper central part of the graph correspond to the responses to stimulus-pairs which contained tones with different timbres and whose order of succession was “congruent” with the direction of ΔF0, i.e., in the case of tones with different F0-s the tone with the higher F0 always had the brighter timbre (hereafter we will call it “the congruential timbral arrangement”). The “hit” rate here is the highest, but the “false alarm” rate is also very high. As a result the *d*′ is lower than in the previous case. Nevertheless, all the data-points are located above the *d*′ = 1 isosensitivity curve. The value *c* < 0 indicates that the bias of the replies was toward giving “false alarms.”

The data-points in the middle central part of the graph correspond to the responses to stimulus-pairs which contained tones with different timbres “incongruent” with the direction of ΔF0, i.e., when in the case of stimuli with different F0-s, the tone with the higher F0 always had the duller timbre (hereafter we will call it “the incongruential timbral arrangement”). Here the “hit” rate is low and the “false alarm” rate is high. The data-points are located close to the *d*′ = 0 isosensitivity diagonal, implying that the ability of participants to tell whether the tones in a given pair had the same or different F0-s was very low and only slightly above chance. The replies were not noticeably biased in either direction as the location of the data-points was at the same time also close to the zero-bias diagonal-line.

Though the general location of the data-points of the responses of pianist and string players at different timbral orders of tones was similar, the *d*′-values of the string players in response to the same paired stimuli were nevertheless always a little higher than those of the pianists, and pianists tended to give “false alarms” more frequently than string players when tones with identical F0-s but with different timbres were compared. The difference in “hit” rates was less clearly pronounced. Generally, the “hit” rate was slightly higher in the case of string players, except in the case of stimuli with different timbres at an “incongruential” timbral arrangement.

In both groups the performance was better (the “hit” rate was higher and the “false alarm” rate lower, and as a result the *d*′-value was higher) when both stimuli in a pair had bright timbres than when they both had dull timbres. In the case of tones with different timbres in the stimulus-pair the participants made fewer “false alarms” when the first tone had a brighter timbre. The effect was more pronounced in the case of pianists.

### d′-values by different ΔF0-s

Table 2 presents the results of the different timbral arrangements of the stimuli broken down by all the investigated ΔF0-s. We can see that: (1) the value of *d*′ is always higher when the ΔF0 is bigger; (2) the value of *c* is always lower (indicating a greater bias toward “false alarms”) at “congruential” timbral order than at “incongruential” timbral order^5^; (3) the *d*′-s of the string players are always higher than those of the pianists (except, at Δ*F0* = 20 cents and at Δ*F0* = −20 cents at “incongruential” timbral orders). We may also note that the average ability of both pianists and string players to detect 20 cents F0 difference at the “incongruential” timbral arrangement of tones used in our experiment is worse than chance, as the value of *d*′ is always below zero in these test conditions. At the same time the value of *d*′ is always well-above *d*′ = 1 (indicating satisfactory sensitivity) when the tones with Δ*F0* = 20 cents and −20 cents have identical timbres.

**Table 2:** **The *d*′- and *c*-values as well as “hit” rates (H) and “false alarm” rates (F) of the responses of pianists and string players, calculated separately for each ΔF0 (the value of which is given in cents) at four different timbral arrangements of tones**.

**Timbral order**		**Pianists**		**String pl**		**Pianists**		**String pl**	
	**Δ*F*0**	***d***′	***c***	***d***′	***c***	***H***	***F***	***H***	***F***
Bright–bright	20	1.73	1	1.92	0.45	0.45	0.03	0.7	0.08
Bright–bright	−20	1.52	1.1	1.65	0.58	0.36	0.03	0.6	0.08
Bright–bright	40	2.52	0.6	2.66	0.08	0.74	0.03	0.9	0.08
Bright–bright	−40	2.44	0.64	2.55	0.13	0.72	0.03	0.87	0.08
Bright–bright	60	3.12	0.3	4.59	−0.89	0.9	0.03	1	0.08
Bright–bright	−60	2.81	0.46	2.95	−0.07	0.83	0.03	0.94	0.08
Dull–dull	20	1.3	1	1.41	0.61	0.36	0.05	0.54	0.09
Dull–dull	−20	1.09	1.11	1.13	0.75	0.28	0.05	0.43	0.09
Dull–dull	40	2.29	0.52	2.66	−0.02	0.74	0.05	0.91	0.09
Dull–dull	−40	2.2	0.56	2.84	−0.11	0.7	0.05	0.94	0.09
Dull–dull	60	2.65	0.34	4.58	−0.97	0.84	0.05	1	0.09
Dull–dull	−60	2.68	0.32	3.75	−0.56	0.85	0.05	0.99	0.09
Bright–dull	20	−0.05	0.15	−0.1	0.36	0.43	0.45	0.33	0.39
**Bright–dull**	**–20**	**0.77**	**–0.26**	**1.1**	**–0.26**	**0.74**	**0.45**	**0.79**	**0.39**
Bright–dull	40	0.48	−0.11	0.6	−0.01	0.64	0.45	0.62	0.39
**Bright–dull**	**–40**	**1.61**	**–0.68**	**1.99**	**–0.71**	**0.93**	**0.45**	**0.96**	**0.39**
Bright–dull	60	1.47	−0.61	2.07	−0.75	0.91	0.45	0.96	0.39
**Bright–dull**	**–60**	**2.16**	**–0.96**	**2.57**	**–1**	**0.98**	**0.45**	**0.99**	**0.39**
**Dull–bright**	**20**	**0.76**	**–0.59**	**0.91**	**–0.28**	**0.83**	**0.58**	**0.77**	**0.43**
Dull–bright	−20	−0.27	−0.08	−0.3	0.33	0.48	0.58	0.31	0.43
**Dull–bright**	**40**	**1.59**	**–1**	**1.82**	**–0.73**	**0.96**	**0.58**	**0.95**	**0.43**
Dull–bright	−40	0.24	−0.33	0.37	0	0.67	0.58	0.58	0.43
**Dull–bright**	**60**	**1.83**	**–1.12**	**3.36**	**−1.5**	**0.98**	**0.58**	**1**	**0.43**
Dull–bright	−60	0.89	−0.66	1.26	−0.45	0.86	0.58	0.86	0.43

*Bold—the timbral arrangement of the stimuli is “congruential” with the direction of* Δ*F0*.

We may conclude that as both the *d*′- and the *c*-values of each group were strongly dependent on the timbral arrangement of the stimuli, the perception of pianists as well as string players was influenced by the timbre-induced pitch shift.

### The distance between *d*′-s and between *c*-s at timbral arrangements with identical and with different timbres: a comparison of the responses of pianists and string players

We can estimate the strength of the timbre-induced pitch shift on the participants' perception by measuring the change in *d*′- and *c*-values when instead of tones with identical timbres tones with different timbres are compared. Corresponding values and changes (distances^6^) in *d*′ and *c* units are presented in Tables 3A,B. We can see that the distance between *d*′-s whose values were calculated from the responses for tones with identical timbres and from the responses for tones with “incongruential” timbral arrangement was larger (but only slightly) in the case of string players. The corresponding distance between *d*′-s based on the responses for stimuli with identical timbres and on the responses for stimuli with a “congruential” timbral arrangement was greater in the case of pianists. Analogous distances between *c*-s were always considerably bigger in the case of pianists (Table 3B). We may conclude that although the responses of pianists and string players were both affected by timbre-induced pitch shift, its impact was a little stronger in the case of pianists.

**Table 3A:** **The distances between *d*′-s based on the responses for stimuli with identical timbres and on the responses for stimuli with “incongruential” timbral order (penultimate column), and between **d**′-s at identical and at “congruential” timbral orders (last column)**. The values of *d*′ and c are presented in the first three columns.

	***d*** ′ identical	***d*** ′ incongruent	***d*** ′ Congruent	**Distance between *d* ′-s identical—incongruent**	**Distance between *d* ′-s identical—incongruent**
Pianists	2.051	0.29	1.21	1.76	0.84
String pl	2.18	0.39	1.49	1.79	0.68

**Table 3B:** **As in Table 3A, but for the distances and values of *c***.

	***c* Identical**	***c* Incongruent**	***c* Congruent**	**Distance between *c*-s identical—incongruent**	**Distance between *c*-s identical—congruent**
Pianists	0.73	−0.19	−0.65	0.91	1.37
String pl	0.27	0.04	−0.52	0.24	0.79

### The impact of the inserted silence interval

**Figure 4:**
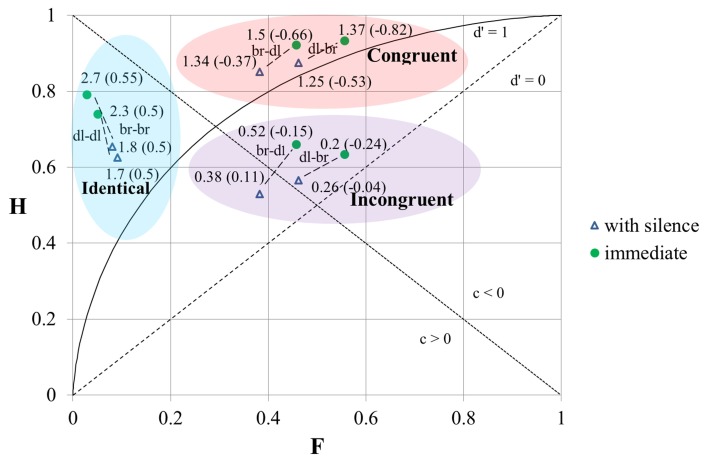
**The results of the SDT analysis of the responses for stimuli with immediate succession and with a 3.5 s silence interval between the individual tones**. H, “hit” rate; F, “false alarm” rate; br, bright timbre; dl, dull timbre. The numbers at the data-points correspond to the values of *d*′ (the first number) and *c* (the number in brackets).

Next, we pooled the responses of all the musicians and presented the results of the SDT analysis for all timbral combinations of tones when they succeeded each other immediately and when a 3.5 s silence interval was inserted between them (Figure 4). We can see that at otherwise similar conditions the value of *d*′ and the “hit” rate were always higher when the stimuli were played in immediate succession (except for the value of *d*′ at an “incongruential” dull–bright timbral sequence of tones). However, the “false alarm” rate was also higher in the case of tones with different timbres played in immediate succession.

Here we also calculated the distances between *d*′-s and between *c*-s, the values of which were based on the responses for tone-pairs at identical timbres and at different timbres (Tables 4A,B). All these distances were smaller when there was a 3.5 s silence interval between the tones. We may conclude that although the sensitivity was higher when the tones succeeded each other immediately, the influence of timbre-induced pitch shift on the responses was nevertheless smaller when there was a brief silence interval between the tones.

**Table 4A:** **The *d*′-values based on pooled replies for the stimulus-pairs containing tones with identical timbres (first column), with different timbres at “incongruential” timbral order (second column), with “congruential” timbral order (third column), as well as the distances between corresponding *d*′-s (in *d*′ units)**.

	***d*** ′ Identical	***d*** ′ Incongruent	***d*** ′ Congruent	**Distance between *d* ′-s identical—incongruent**	**Distance between *d* ′-s identical—congruent**
Immediate	2.44	0.36	1.45	2.08	0.99
With 3.5 s	1.65	0.32	1.29	1.33	0.36

**Table 4B:** **As in the Table 4A, but for the distances and values of *c***.

	***c* Identical**	***c* Incongruent**	***c* Congruent**	**Distance between *c*-s identical—incongruent**	**Distance between *c*-s identical—congruent**
Immediate	0.54	−0.2	−0.74	0.73	1.28
With 3.5 s	0.54	0.04	−0.45	0.5	0.99

*The data is presented separately for the replies at immediate succession of comparable tones (first row) and for the replies when the silence interval with a duration of 3.5 s was inserted between the tones (second row)*.

### The impact of the pitch region

**Figure 5:**
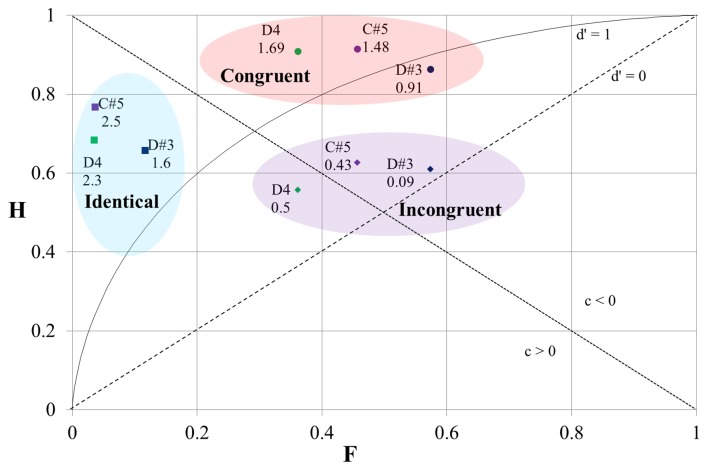
**The results of the SDT analysis of the pooled responses for stimuli by three different pitch regions (around D#3, D4, and C#5)**. The location of the data-point on the graph depends on the timbral arrangement of the comparable tones (the three colored ovals). The numbers on the graph correspond to the values of *d*′.

The results of the SDT analysis of the pooled responses presented by the three pitch regions (at D#3, D4, and C#5) are shown in Figure 5. We can see that the location of the data-points at three distinct areas (*identical/congruent/incongruent*) depends on the timbral arrangement of the stimuli in a similar way in the case of all three pitch regions. The more precise location inside the areas, however, still seems to depend somewhat on the pitch region. For example, the value of *d*′ was always noticeable lower at D#3 compared with the other pitch regions. This may be caused by the differences in frequency DL which, for pure tones, is smallest at middle frequencies at around 500 Hz (Sek and Moore, 1995) and from which D#3 is furthest away.

### Individual *d*′-s at different timbral arrangements

We also calculated the individual values of *d*′, *H*, and *F* for all four timbral combinations of the stimuli. Although the number of replies given by each single participant was much smaller than the number of pooled responses for the whole group, meaning that the power to characterize an individual person's perception objectively is weaker, nevertheless such an analysis allows us to obtain some tentative information with regard to interpersonal variability.

**Figure 6:**
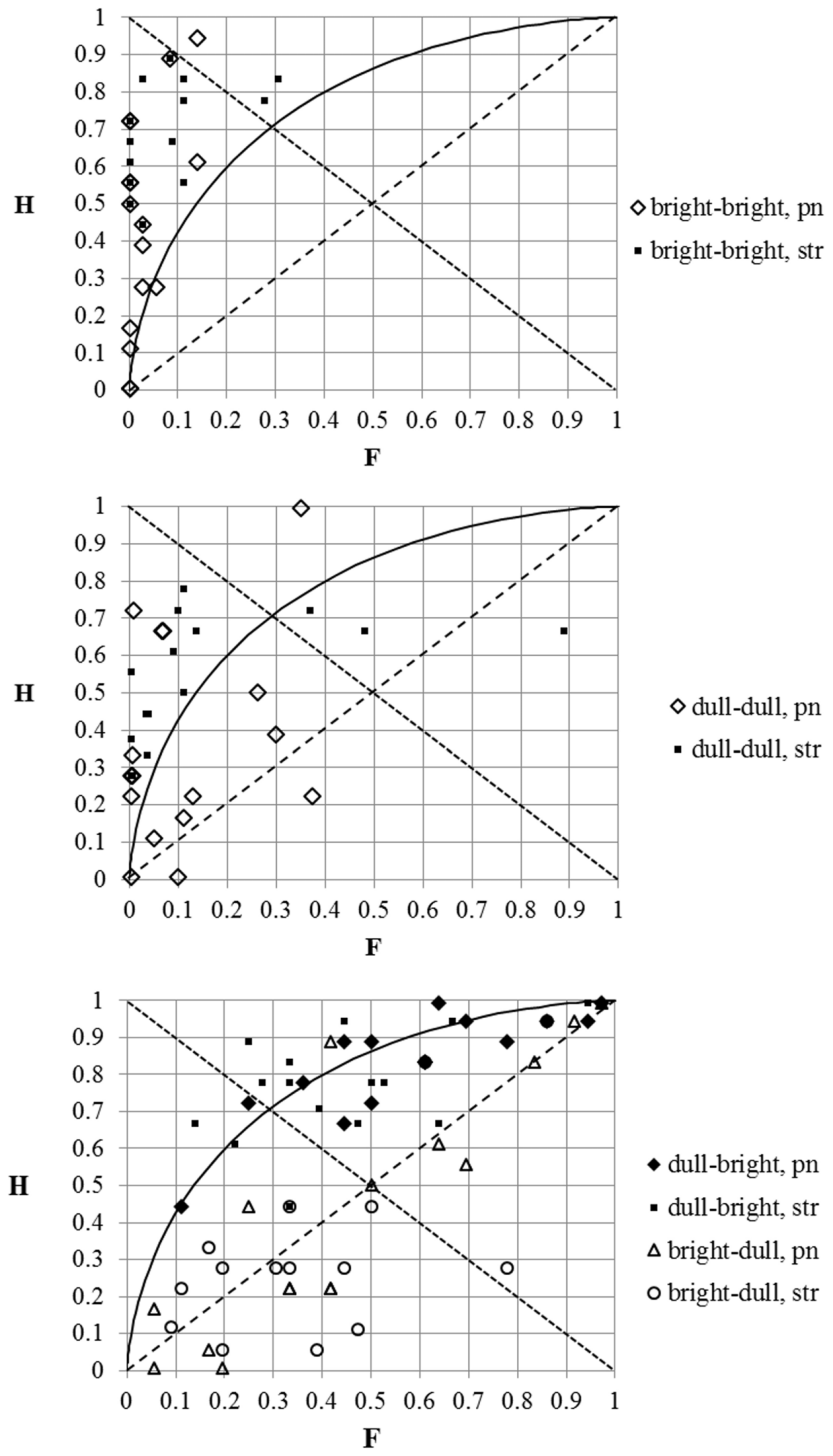
**Individual data-points of the SDT analysis at different timbral arrangements of comparable tones (see separate panels)**. Diamonds—the data-points of pianists; squares—the data-points of string players. Only the replies to the stimulus-pairs with Δ*F0* = 20 cents and zero cents were included in the analysis.

First, let us look at the individual responses at Δ*F0* = 20 cents when both stimuli had bright timbres (Figure 6, upper panel). The majority of data-points are located inside a triangle on the left side of the graph where *c* < 0 (indicating a bias to “miss” the “signals”). This tendency was especially strong in the case of several pianists (see the empty diamonds in the lower left corner of the graph) and the “hit” rate of their responses was correspondingly low. One of these pianists “missed” all the “signals” (which also corresponds to a zero “hit” rate). Although the “false alarm” rate here was almost always below about 15%, in the case of two string players it was as high as around 30%. Six participants (four string players and two pianists) were able to “hit” more than 80% of the “signals.” The highest personal sensitivity reached Max *d*′ = 3.36 (Average *d*′ = 2.14, *SD* = 0.88, Min *d*′ = 0.23).

When both tones in the stimulus-pair had the same dull timbre, the performance of the participants was slightly less good (see Figure 6, middle panel). While in the case of a bright–bright arrangement of tones only two participants had *d*′ < 1, here the number increased to 11, and for three of these the *d*′-value was even below zero. The “false alarm” rate was low (it was below 15% for more than half the participants, although in the case of one of the string players it was as high as 89%). The highest individual sensitivity reached Max *d*′ = 2.95 (Average *d*′ = 1.31, *SD* = 1.07, Min *d*′ = −1.26).

There are two extreme listening modes for complex tones. In the case of spectral mode, the listeners are decomposing the sound to single partials. For “spectral listeners” the pitch of a complex tone is based on the pitch of its single partial (i.e., on its F0 component). The holistic mode relies on the acoustic waveform as a whole (and the two most recognized hypotheses describing the process in detail are the *autocorrelation* and the *pattern matching*). (Schneider and Wengenroth, 2009; de Cheveigné, 2010) The lower *d*′ of our participants in the case of dull–dull tone-pairs could be explained by the possibility that they had a higher propensity to the spectral listening mode for the tones with dull timbre because of the dominance of the F0 component in these tones. Lower values of *d*′ are expected as the pitch JND of sine tones below 2 kHz tends to be somewhat higher compared to complex tones at the same frequency of oscillation (Henning and Grosberg, 1968).

The “hit” and “false alarm” rates changed significantly in the case of all the participants when the tones compared had different timbres (see Figure 6, lower panel). Now the data-points are located either in the upper triangle of the graph, where *d*′ > 0 and *c* < 0 (indicating a bias to make “false alarms”), or in the lower triangle, where *d*′ < 0 (indicating a lower than chance ability of the participant to discriminate “signals” from “noise”) and where *c* > 0 (indicating a bias to “miss” “signals”). In the first case the timbral sequence of the tones was “congruential” with the direction of ΔF0, while in the second case it was “incongruential.” The average of individual “false alarm” rates increased to 42% at a bright–dull timbral arrangement and to 51% at a dull–bright timbral arrangement of tones.

The individual *d*′-values were noticeably higher at a “congruential” timbral arrangement (Average *d*′ = 0.95, *SD* = 0.5, Max *d*′= 2.18, Min *d*′ = 0) than at an “incongruential” timbral arrangement (Average *d*′ = −0.15, *SD* = 0.7, Max *d*′ = 1.43, Min *d*′ = −1.68; generally, the sensitivity values were here never higher than *d*′= 0.69, except in the case of one pianist).

In the case of Δ*F0* = 60 cents (see Figure 8) almost all the data-points are located in the upper triangle of the graph where *c* < 0 (indicating a bias to “false alarms”). The “hit” rate was close to 100% in the case of the string players when tones had identical timbres or when the timbral arrangement of the tones was “congruential” with the direction of Δ*F*0. It is interesting to note that in the case of some pianists the “hit” rate was still very low, even when the tones compared had identical timbres. At an “incongruential” timbral arrangement of the stimuli the “hit” rate in the case of several participants was substantially lower. Nevertheless, the data point for the majority of the participants lay above the *d*′ = 1 curve, and no participant's individual sensitivity was ever below *d*′ = 0.35.

**Figure 7:**
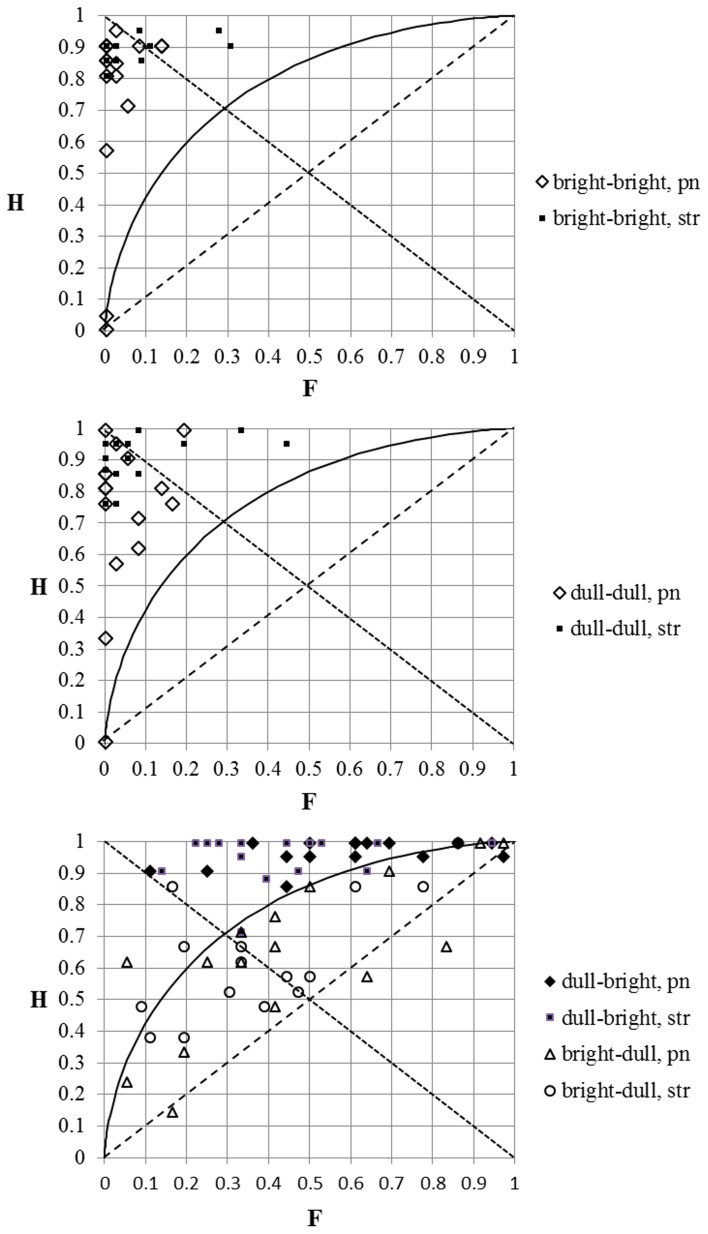
**The same as Figure 6, but only the replies to the stimulus-pairs with Δ*F*0 = 40 cents and zero cents were included in the analysis**.

The tendencies of the responses for tone-pairs with Δ*F0* = 40 cents (see Figure 7) were in the middle of those described at Δ*F0* = 20 cents and at Δ*F0* = 60 cents. Most data-points at bright–bright and dull–dull timbral arrangement were located below the *c* = 0 diagonal, indicating the bias to “miss” signals. Most of the data-points at an “incongruential” timbral arrangement (Figure 7, bottom panel) were located between the *d*′ = 0 diagonal and the *d*′ = 1 curve, indicating a poor ability—but still greater than chance—to detect the tone pairs with an F0 difference.

Here we have reported only the results based on the responses for tone pairs where the F0 of the second tone was the same or higher than the ΔF0 of the first tone. As the results were analogous at negative Δ*F0*-values (i.e., when the second tone in the pairs had a lower F0 compared to the first tone), these are not presented here.

### Individual gross *d*′-s based on pooled replies over all test variables

Our final step was to calculate the individual *d*′, *H*- and *F*-values based on pooled replies over all the test variables (see Figure 9). In order to give a better overview we have circled in freehand the areas which include the data-points of pianists and the data-points of string players. We can see that both areas almost overlap at their upper margins where *d*′−values are high. The area of the pianists' responses extends downwards toward lower *d*′-values, implying that the variability in *d*′ of the pianists was greater compared to that among the string players, and that someone with a low *d*′-value is more likely to be a pianist than a string player.

## The statistical significance of the results

The SDT does not allow us directly to assess the statistical significance of the difference in *d*′ at different test conditions. Nevertheless, we can use two indirect methods for this purpose. The first of these is based on the so called “rule of eye,” according to which the difference in the statistical mean of any two items is significant at *p* ≤ 0.05 if in their “bar chart” comparison their standard error (SE) bars do not overlap, and if the gap between the ends of the corresponding SE bars is at least about the size of the average SE (Cumming and Finch, 2005). In our case we can compare the *d*′-s in such a way. We calculated the *SE*-values which, for the *d*′-s in Figures 3–5, were all between 0.05 and 0.1, and for the *d*′-s in Table 2 between 0.09 and 0.2. Further comparison by the “rule of eye” allowed us to conclude that at *p* ≤ 0.05 the following differences in *d*′ were statistically significant: (1) between that of the pianists and the string players, based on the pooled replies over the all test conditions as presented in Table 1; (2) between that at identical, congruential and incongruential timbral orders at otherwise the same test conditions, as presented in Figures 3–5 and in Table 2; (3) between that of the pianists and the string players at congruential timbral order, as presented in Figure 3; (4) between that of the pianists and the string players in the case of the following rows in Table 2: bright—bright at Δ*F*0 60 cents, dull—dull at Δ*F*0 −40, 60, and −60 cents, bright—dull at Δ*F*0 −20, −40, 60, and −60 cents, and dull—bright at Δ*F*0 60 cents; (5) between that at the condition *with silence interval—immediate* in the case of identical timbres of the tones, as presented in Figure 4; and (6) between that at pitch regions D#3—D4 and D#3—C#5 in the case of all three timbral combinations (identical, congruential and incongruential), as presented in Figure 5. Still, the “rule of eye” method can give only rough estimates of effects, but not of interactions.

The second possibility to assess the statistical significance of the results is to conduct a repeated measures ANOVA test on the individual *d*′-s. Here the main problem is the impreciseness of the *d*′-values, as the number of tone-pair repetitions for “hit” and “false alarm” rate calculations at each combination of test conditions was small. In order to increase the preciseness we decided to conduct two separate ANOVA-s where we pooled the replies of a participant in two different ways. This allowed us to obtain *d*′-values whose SE-s all remained between the values of 0.4 and 0.7.

**Figure 8:**
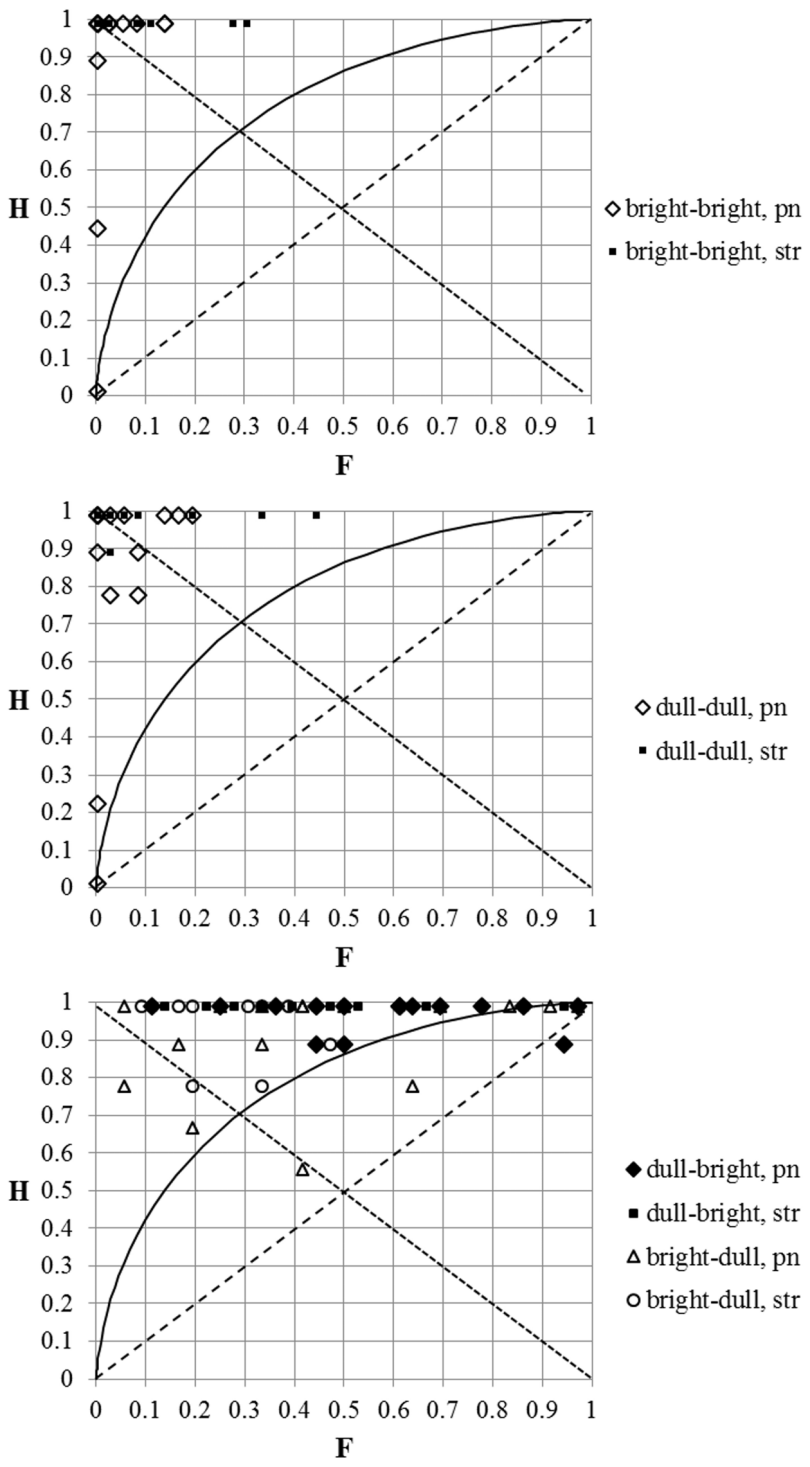
**The same as Figure 6, but only the replies to the stimulus-pairs with Δ*F0* = 60 cents and zero cents were included in the analysis**.

For the first ANOVA test we used the *d*′-s which were based on the pooled replies over all three pitch regions and over the test conditions with and without a silence interval and which correspond to the data-points in Figures 6–8. The within-subjects factors of the first ANOVA test were: (1) the *timbral order* (br–br/dl–dl/br–dl/dl–br), (2) the *direction of the F0 change* (up/down), and (3) *|*Δ*F*0*|*; the between-subject factor was the *instrument* (pn/str). The test showed that the two strongest impacts were from |Δ*F*0|[*F*_(2, 58)_ = 229.91, *p* = 0.000, partial η^2^ = 0.89], and from the *timbral order* [*F*_(3, 87)_ = 93.33, *p* = 0.000, partial η^2^ = 0.76]. (We do not include here any average values of *d*′, as these were close to the group values described earlier in Table 2.) Also the influence from the *direction of the F0 change* was statistically significant [*F*_(1, 29)_ = 5.17, *p* = 0.03, partial η^2^ = 0.15]. Likewise, the following inter-factor interactions were statistically significant: (1) between the *timbral order* and the *direction of F0 change* [*F*_(3, 87)_ = 100.87, *p* = 0.000, partial η^2^ = 0.78]; (2) between the *timbral order* and |Δ*F*0|[*F*_(6, 174)_ = 24.43, *p* = 0.000, partial η^2^ = 0.46]; (3) between the *direction of F0 change* and|Δ*F*0|[*F*_(2, 58)_ = 3.46, *p* = 0.04, partial η^2^ = 0.12], (4) between the *timbral order*, the *direction of F0 change* and |Δ*F*0|[*F*_(6, 174)_ = 16.35, *p* = 0.000, partial η^2^ = 0.36] and (5) between the *timbral order*, |Δ*F*0|and the *instrument* [*F*_(6, 174)_ = 3.84, *p* = 0.001, partial η^2^ = 0.12].

**Figure 9:**
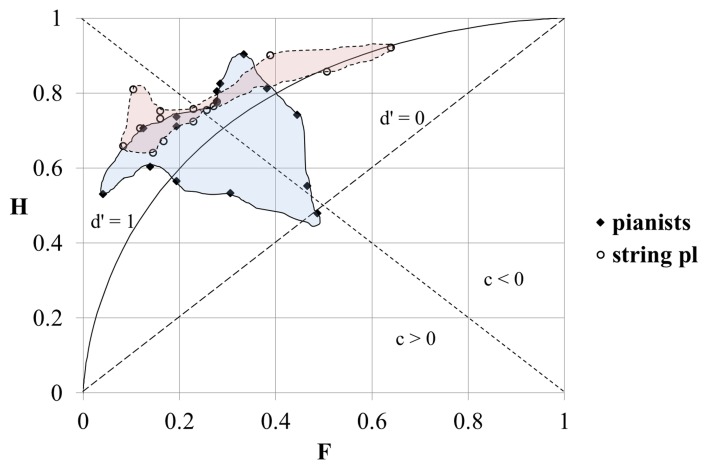
**The individual overall sensitivities of participants based on pooled replies over all test variables**. The hand-drawn shapes incorporate the individual data-points of pianists (blue area) and string players (pink area). H, “hit” rate; F, “false alarm” rate.

For the second ANOVA test we pooled the replies over all Δ*F*0-s and examined the significance of the influence on *d*′ from (1) the *timbral congruency* (identical/congruent/incongruent), (2) the presence/lack of the *silence interval*, and (3) the *pitch region* of the tones (D#3/D4/C#5). Also here the between-subject factor was the *instrument* (pn/str). The test showed the biggest impact from the *timbral congruency* [*F*_(2, 726)_ = 166.98, *p* = 0.000, partial η^2^ = 0.8]. The factor *pitch region* yielded an *F* ratio of *F*_(2, 58)_ = 30.0, *p* = 0.000, partial η^2^ = 0.51. The impact from the factor *silence interval* was statistically significant, but only marginally so [*F*_(1, 29)_ = 3.23, *p* = 0.08, partial η^2^ = 0.1]. Also the second ANOVA test, too, showed that some interactions between the factors were statistically significant. First, between the *timbral congruency* and the *silence interval* [*F*_(2, 58)_ = 11.67, *p* = 0.000, partial η^2^ = 0.29]; second, between the *silence interval* and the *pitch region* [*F*_(2, 58)_ = 10.42, *p* = 0.000, partial η^2^ = 0.26] and third, between the *timbral congruency*, the *silence interval* and the *pitch region* [*F*_(4, 116)_ = 6.18, *p* = 0.000, partial η^2^ = 0.18]. Both ANOVA-s were not reliable to investigate the significance of the impact from the *instrument* because of the low observed power of the tests (correspondingly 0.24 and 0.33).

To conclude, the methods described above—the “rule of eye” for the comparison of *d*′-s together with their SE bars and the use of ANOVA tests for individual *d*′-values—showed similarly (or complementing each other) that the main findings of this study concerning the *d*′, are statistically significant.

## Discussion and conclusions

In this research we used the SDT paradigm in order to investigate the timbre-induced pitch shift phenomenon in the pitch comparison of musical tones with tiny F0 differences. Our analysis revealed that the sensitivity (*d*′) of participants to detect F0 difference in pairwise consecutively played tones deteriorated and the bias of their replies changed from “missing” the “signals” toward making “false alarms” when instead of tones with the same timbres the comparable tones had different timbres. The deterioration in *d*′ was present in the perception of all the 16 pianists and 15 string players tested, and it was greater when the tones with a higher F0 in the pair had a duller timbre. In the case of those tones with dull and bright timbres used in our experiment, on average the professional musicians in our study were not able reliably (with *d*′ ≥ 1) to discern a 40-cent F0 difference when the tone with the higher F0 had a duller timbre. At an individual level, none of the participants (with the exception of one pianist) could reliably detect a 20-cent F0 difference in the same conditions, though some of them were still able to detect a 40-cent F0 difference. However, most of the participants successfully identified a 20-cent F0 difference when the tones had the same timbre, and especially when the timbre of the tones was bright. Our perception experiment also showed that the timbral difference, which is able to elicit the effect just mentioned, can only be based on the difference in the relative levels of the fundamental spectral components of the paired tones.

Although the effect described above was present in the perception of all the musicians tested, there were still substantial individual differences. On average, the *d*′ of string players was higher than that of pianists, and both the *d*′- and the *c*-values among the string players were less influenced by the difference in the timbral arrangement of tones than in the case of the pianists under the same test conditions. The highest *d*′-values of pianists and string players were quite similar. A bigger difference between the two groups was registered at the lower end of the *d*′-values, where those with low sensitivity tended to be pianists rather than string players. The responses of some of the participants (mostly pianists) were almost always based on the timbral and not on the F0 difference of the tones, even at Δ*F0* = 60 cents. Therefore, we may conclude that the specific type of long-term musical practice undertaken may have some impact on the musician's sensitivity to discriminate small pitch differences. Although the type of musical instrument practiced by the musician over a long period of time may also influence the degree of vulnerability to timbre-induced pitch shift, such vulnerability still tends to remain present even if the instrument concerned (the musician's main instrument) needs continuous pitch adjustments during performance. We still cannot rule out the possibility that children with better pitch perception ability prefer stringed instruments to the piano when they begin their music studies, and that this possible difference in their pitch discrimination abilities does not disappear in their adulthood. String players may also have been in a somewhat better position compared to pianists in our perception test because the viola-like sounds which were used in the experiment were perhaps more familiar to them than to the pianists.

Seven of our participants possessed AP. Their results did not differ noticeably from the results of the other participants, and the variability in their individual *d*′-values was quite similar to the variability of the whole group. However, the experiment should be carried out with a larger number of participants with AP in order to form more certain conclusions.

The silence interval between comparable tones also has some influence on perception. On average the participants demonstrated a higher sensitivity to discriminate pitch differences when the tones were presented in immediate succession (i.e., within the time frame of echoic memory processes) than when a 3.5 s silence interval was inserted between comparable tones (so that short-term memory mechanisms were activated). This can be explained by the increase of “memory noise” which characterizes the tendency of any sensation to be “forgotten” with the passage of time (Demany et al., 2005) as the retention of sensory information is not as effective as the rehearsing with categorical information (Kaernbach and Schlemmer, 2008). However, the rate of “false alarms” started to increase in the case of the immediate succession of tones when the stimuli had different timbres, and as a result the difference in the timbral arrangement of tones had more influence on the values of *d*′ and *c* than when the tones had a 3.5 s silence between them. This implies that vulnerability to timbre-induced pitch shift is higher when the tones are presented in immediate succession, and that our nervous system requires some time in order better to separate pitch information from timbral information.

When designing our perception test we tried to avoid the influence of the random sensation of tonality from adjacent stimulus-pairs; for this reason the F0 of the stimuli was kept random. However, the present design of the experiment could also have caused problems. It is possible that random memory glimpses from adjacent stimulus-pairs might still have disturbed the responses of participants to some extent, especially when there was a silence interval between comparable tones. This could explain the deterioration in the sensitivity of participants in the case of the presence of the silence interval. We may speculate that if we had used fixed F0-anchor-points (i.e., if the first tones in the pairs had always had the same F0-s), the higher *d*′-s would have been registered not in the case of the immediate succession of tones, but when the silence interval was present, because during that time the participants would have tried to recall the anchor-point's pitch by using their short-term or even long-term memory processes in order to strengthen its aural imagination. (At the same time the impact of such processes has to overcome the detrimental influence of “memory noise.”) The results of our experiment also showed that the phenomenon of timbre-induced pitch shift exists quite uniformly, at least at those pitches lying in the region around an octave either side of C4, which are those used most frequently in Western music.

What are the implications of the findings of the present work for music practice? Tones with different timbres, which is the prerequisite for timbre-induced pitch shift, are common in ensemble and orchestral music. The timbre of sounds from one musical instrument may also differ somewhat at different pitch regions and at different volume levels. Likewise, the timbre of singers' voice varies when different vocal techniques are used and also because of the variation of phonemes when uttering the text.

In our experiment the pitch of two tones was compared outside a musical context. This is similar to the pre-performance tuning of musical instruments where the goal of the musicians is to equalize the F0 of the tone produced by their instrument with the F0 of the reference tone (which is usually played by the first violinist or oboist in the case of symphony orchestras). The suggestion from the results of our work is to sound both these tones simultaneously (which is usually the case in the practice of musicians). This allows musicians to use sensory consonance (the cessation of beats) as the cue. In the case of a sequential execution of the tones, the possible timbre-induced pitch shift could lead to smaller or larger F0 mismatches.

It is not so straightforward to predict the implications of the results of this work on the performance of music. For this, further more specific experiments would be required. Furthermore, the results of our experiment are not directly transferable to the comparison of arbitrary sounds with different timbres or to every musical situation. Nevertheless, our findings seem to support the viewpoint expressed, for example, by Morrison and Fyk (2002), namely that good intonation is the matter of compromise and negotiation rather than conformity to some theoretical tuning system. There are also several other factors besides the timbre-induced pitch shift which may induce musicians to depart from the *F*0-values of the established theoretical tuning system (while such deviation is recognized as acceptable intonation by audiences). For example, Sundberg et al. (2013) have shown that professional opera singers may use pitch deviations (which are sometimes even bigger than 50 cents, but which are not usually heard by listeners as pitch effects) for expressive purposes. A similar tendency to use intonation for expressive purposes by string quartet members has been reported by Johnson (2004). In addition, several investigations (Rakowski, 1990; Burns, 1999; Rosner, 1999) have revealed that musicians tend to compress small pitch intervals and enlarge big intervals, and that such deviations are similarly preferred by the listeners. Moreover, listeners tend sometimes to perceive the intonation of the different instruments in multi-part music somewhat independently of each other and to ignore small tuning differences between them (e.g., between vocal solo and piano accompaniment; Sundberg et al., 1996).

We should also not forget that musicians always have some freedom in choosing the *F*0-value of any performed note without losing its scale step identity, because the perception of music intervals tends to be categorical. A similar difference in interval size is less noticeable when both intervals stay within that interval's category limits as opposed to when they are located on different sides of the border between two categories (Burns, 1999).

If good intonation depended only on subjective pitch height, biased as it is by timbre-induced pitch shift, and if that pitch height were perceived similarly by all listeners, then musicians could simply adjust the F0 of executed tones by a factor whose value would depend on the timbre of the tone in order to compensate for that pitch shift. However, using such a correction would not guarantee good intonation for several reasons. First, some partials of a tone can be masked by the tones from other musical instruments. Such masking changes somewhat the timbre of the tone. Still, the level of masking, and hence the timbral change, may depend on the location of the listener—the effect of masking may be absent for someone standing close to the instrument that is masked (e.g., for the player him/herself), and masking can be strong for those persons who are close to the masking instrument. Secondly, the degree of vulnerability to timbre-induced pitch shift is idiosyncratic—the same correction may not be optimal for everybody. Finally, the sensory dissonance factor comes into play when corresponding tones with different timbres are played simultaneously (although in the case of tones with vibrato the importance of sensory dissonance diminishes as the beats are blurred).

Timbre-induced pitch shift may also be among the reasons why the novice opera singers sometimes find it difficult to sing in tune. It is known that the timbre of the voice is different for the singer him/herself compared with how it sounds to others, as for the singer an essential part of his/her own voice reaches his/her hearing system via bone conduction (the transfer function of which is different from the air's transfer function), and because of the triggering of the stapedius reflex, which also affects how a singer perceives the timbre of his/her own voice (Vurma, 2013).

We may speculate that timbre-induced pitch shift could well be a factor which plays a role in the bad timbral blending of the tones of some musical instruments. Sandell (1995) has shown that the similarity of spectral centroids (or the similarity in brightness, as these two are strongly correlated) is one of the most important factors for a good blend. We may speculate that in the case of poor blending there is a conflict between the pitch match based on the equality of F0-s and the pitch match based on the equality of pitch height (which is affected by the timbral difference of the tones). We may encounter similar blending problems in vocal ensembles and choir voice sections when the voice timbre of the singers is noticeably different. As a rule, in multi-part vocal music the sung text is usually organized in such a way that the same vowel sounds (i.e., the tones with the same timbre) are always sung together, at least on those notes which have longer durations, as this also supports better blending.

In conclusion, the results of this paper support the findings of some of the previous works listed in the introduction, which suggest that timbre and pitch are interacting parameters and that the timbral difference of musical tones can considerably bias the judgments of even professional musicians when they have to compare the pitch of consecutively played sounds. In real musical performances we have to accept that good intonation can be sometimes ambiguous and that among the factors which influence it is the aural illusion of timbre-induced pitch shift.

### Conflict of interest statement

The author declares that the research was conducted in the absence of any commercial or financial relationships that could be construed as a potential conflict of interest.

## Footnotes

^1^Beats are the undulation in the amplitude of a pooled sound wave consisting of two sounds with slightly different frequencies of oscillation, because of the changing phase relationships between them. The rate of the undulation equals the frequency difference between the two tones. Beats can also emerge between the upper partials of the tones.

^2^ΔF0 = F02 – F01, where F01 and F02 are the fundamental frequencies of the first and second tone in the pair.

^3^In order to obtain the tones with a dull timbre we suppressed the whole frequency range of the original tone's spectrum above the F0 component by about −6 dB; for the tones with a bright timbre we suppressed only the F0 component by about −30 dB. Finally, the general sound pressure levels of the tones was equalized.

^4^The pitch correction algorithm which is used in WaveLab5 software is a trade secret. However, the selection of the type of algorithm was not critical, since in our experiment we had single pitched monophonic tones (whose processing is less vulnerable to artefacts) and the necessary pitch correction was less than 3%.

^5^Although the “false alarm” rate was the same for both timbral orders (as the tone pairs with identical F0-s always remained the same), nevertheless the bias of the replies toward “false alarms” increased as it reflected the tendency of more “hits.”

^6^The mathematical property of *d*′ as a distance measure has been described by Luce (1963).
